# The Fine Old Irish Surgeon*From an early issue of the “Red Back.”

**Published:** 1911-11

**Authors:** 


### The Fine Old Irish Surgeon.*

(Air: The Fine Old English Gentleman.)

BY THE EDITOR.

There was a fine old Irish surgeon from Erin's Emerald Isle,
Who dashed around in a spanking double team in the biggest kind
of style.
His reputation as an operator extended far and wide,
And patients by the hundreds flocked to see him in an ever swelling
tide,
This fine old Irish sur-ge-on, from Erin's Emerald Isle.

*From an early issue of the "Red Back."

This fine old Irish surgeon was a man of wondrous guile,
Who by the practice of surgery and sundry other arts had managed to accumulate quite a respectable little pile.
He had a perfect mania for operating, and a fee he never missed;
For sufficient pecuniary consideration he'd tackle anything on God's green earth, from a bone felon to a four hundred-pound firmly adherent multilocular ovarian cyst,—
This fine old Irish sur-ge-on, from Erin's Emerald Isle.

This fine old Irish sur-ge-on, his leisure to beguile,
Would tell the darndest, toughest, most highly improbable, Munschausen-like surgical yarns and never crack a smile;
He didn't care a continental for his patients' souls,—that was none of his business; for their bodies he cared still less,
And although most of his patients died on the operating table, and the others immediately after being removed, he'd claim, with more truth than poetry that every operation was a "bloody big success,"
This fine old Irish sur-ge-on, from Erin's Emerald Isle.

One day there came a woman, whose baby, she related,
In consequence of a grain of corn firmly impacted in the trachea was well nigh asphyxiated.
"Och, Doctor, dear, it's dead he is, as sure as ye's are born,
Unless be the holy mother ye's can extricate the murtherin' grain o' corn,
Ye's fine old Irish sur-ge-on, from Erin's Emerald Isle."

"Be aisy, now," the doctor said, "and never ye's moins a whinnet;
A sample of me wondrous skill I'll show ye's in a minit,—
Tracheotomy, I'll perform, the sixteenth case today (moind *that* now),
And be the holy powers, while ye wait, that same pesky little grain o' corn I'll quickly bring away," said this fine old Irish sur-ge-on in his grandi-loquent style.

The baby then he quickly took,—now in *articulo mortis,*
And laid it on the operating table, then just as quickly brought his instruments, and cutting down, through one tissue then another, he did not stop till he had opened the trachea, *secundem artem,* and with a pair of Gross' latest improved alligator forceps seized the offending foreign body and hastened in triumph to show it to the anxious waiting mother.
This fine old Irish sur-ge-on, from Erin's Emerald Isle.

"Och, madam, dear, here's the grain o' corn,—I got it, you just bet.
Faith, it's meself's the man to do the thing to which me hand is set.
The *baby?* oh, the baby,—(I think it was, ye said?)
I got the murtherin' grain o' corn, I did, but the baby, marm, is dead!" said this fine old Irish surgeon, with his most complacent smile.

---

THE SOUTHERN MEDICAL ASSOCIATION had a splendid meeting at Oklahoma City recently. Dr. Clark (El Reno, Okla.), the popular secretary, sent us a full report of it, but it came too late for use in this issue, and would be a back number in December. We regret this very much, as many of my readers are members and attended the meeting and would have been glad to see the report in their favorite journal, the "Red Back."

